# Bionic Design, Materials and Performance of Bone Tissue Scaffolds

**DOI:** 10.3390/ma10101187

**Published:** 2017-10-17

**Authors:** Tong Wu, Suihuai Yu, Dengkai Chen, Yanen Wang

**Affiliations:** 1 Shaanxi Engineering Laboratory for Industrial Design, Northwestern Polytechnical University, Xi’an 710072, China; wutong@nwpu.edu.cn (T.W.); ysuihuai@vip.sina.com (S.Y.); chendengkai@nwpu.edu.cn (D.C.); 2 School of Mechanical Engineering, Northwestern Polytechnical University, Xi’an 710072, China

**Keywords:** bone tissue scaffolds, bionic design, materials, performance

## Abstract

Design, materials, and performance are important factors in the research of bone tissue scaffolds. This work briefly describes the bone scaffolds and their anatomic structure, as well as their biological and mechanical characteristics. Furthermore, we reviewed the characteristics of metal materials, inorganic materials, organic polymer materials, and composite materials. The importance of the bionic design in preoperative diagnosis models and customized bone scaffolds was also discussed, addressing both the bionic structure design (macro and micro structure) and the bionic performance design (mechanical performance and biological performance). Materials and performance are the two main problems in the development of customized bone scaffolds. Bionic design is an effective way to solve these problems, which could improve the clinical application of bone scaffolds, by creating a balance between mechanical performance and biological performance.

## 1. Introduction

### 1.1. Bone-Tissue Engineering

Tissue Engineering (TE) is a new interdisciplinary subject, established in the 1980s. Its fundamental purpose is to construct artificial substitutes with biological function in vitro, based on the application of basic principles and techniques of engineering and life science [1], for the repair and regeneration of damaged tissues and organs, or to replace part, or all, features of the failing tissues or organs [2]. Bone-tissue engineering is an important part of TE.

**Figure 1:**
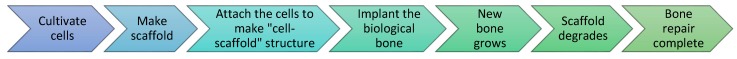
Bone tissue repairing process.

Bone tissue engineering repair or reconstruct bone defects by combining biomaterials, cells, and signaling factors [3]. The repair or reconstruct process is: (1) cultivate cells and make scaffold; (2) inject the cells into the scaffold, make “cell-scaffold” structure; (3) implantation of the structure; (4) new bone grows and scaffold degrades; and, (5) bone repair complete [4,5]. A more detailed process of bone tissue repairing is shown in Figure 1.

In general, bone-tissue engineering consists of three basic elements, namely the cells, the growth factors (such as bone growth factor, bone inducing factor, etc.), and the bone scaffolds (extracellular matrix of biological material, required for cellular proliferation and differentiation) [6,7].

As a framework for bone tissue regeneration, the scaffold material directly affects the biological characteristics of seed cells, as well as the survival, migration, proliferation, and metabolism of the cells. Therefore, it is a key element in bone-tissue engineering [8].

Bone scaffolds not only match the shape of the replaced bone, but also have a three-dimensional microstructure that provides channels for tissue growth, differentiation, nutrition delivery, and metabolism, while also being sufficiently strong to provide mechanical support.

### 1.2. Anatomical Structure and Mechanical Performance of Biological Bone

Biological bone is mostly composed of inorganic mineral salts and organic biological proteins. The inorganic mineral salts in bone tissue, such as hydroxyapatite (HA), account for about 60% to 70% of the total dry bone weight. Organic proteins, represented by type I collagen (approximately 95%), chondroitin sulfate, keratin, and amorphous matrix, etc., account for the remaining 30–40% of the dry bone weight [9]. From the perspective of material science, biological bone can be considered a composite material, mostly formed of HA and type I collagen [10].

**Figure 2:**
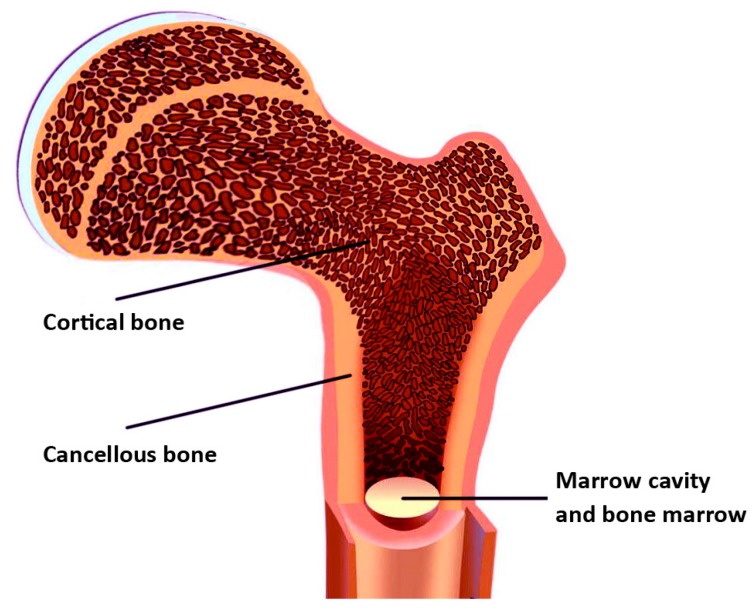
The anatomical structure of biological bone.

The anatomical structure of biological bone tissue consists of cortical bone, cancellous bone, and bone marrow cavity (shown in Figure 2). This structure provides the basis for the development of the bionic bone model. The mechanical performance of cortical bones is very good. However, the mechanical performance of cancellous bone may vary, due to important anatomical differences. The compressive strength of the cortical bone is 170–193 MPa, whereas that of cancellous bone is 7–10 MPa [11]. Therefore, the scaffold model of personalized bionic bone must be designed and processed according to the structure of the real bone, and should take into account the mechanical and biological properties of each component, to ensure the stability of the implant [12].

## 2. Performance of Materials Used in Scaffolds Designed for Bone Regeneration

### 2.1. Biocompatibility

Biocompatibility implies no toxicity, no teratogenicity, no inflammatory reaction, no hemolysis, and no coagulation reaction, etc., meaning that a material can be safely used in the human body. Particularly regarding degradation, a scaffold material ought to support normal cellular activity, without being toxic to the host tissue [3]. Implanted in the body, the material should not be repelled or cause chronic infection. Moreover, the material should not be cytotoxic to newly formed tissue/surrounding host tissue or display systemic cytotoxicity [13,14].

The bone scaffold material should be conducive to cell adhesion, and provide a good micro-environment for cell growth. It is always desirable to chemically combine the scaffold with bone and with biological activity.

In addition, the degradation of scaffold material is another important consideration [15]. The material should be biodegradable, to allow for bone ingrowth over time [16].

### 2.2. Osteoinductivity and Osteoconductivity

In addition to good biocompatibility, the synthetic bone scaffold should simultaneously have good osteoinductivity and osteoconductivity. Osteoinductive bone scaffold materials, in combination with growth factors, promote the proliferation of bone marrow mesenchymal stem cells (MMSCs), as well as their differentiation into osteoblasts after implantation. The three-dimensional structure and porous matrix of the bone scaffold with good osteoconductivity, enable the growth of the bone tissue along its surface or internal pores. The newly formed osteoblasts in bone scaffolds with good osteogenesis have the potential for further mineralization and formation of new external bone matrix.

Therefore, many scholars have improved the osteoinductivity of bone scaffolds, by changing their physical and chemical properties or by incorporating bone supplements into the scaffolds [17]. Moreover, the porosity and pore structure of bone scaffolds can be adjusted to enhance the osteoconductivity [18,19].

### 2.3. Porosity, Pore Diameter and Pore Structure

Ideal bone scaffolds should have interconnected pore networks to promote oxygen, nutrient and waste exchange [5]. This structure plays a decisive role in the osteogenesis of seed cells. The microscopic pore structure of the scaffold mainly refers to porosity, pore size, pore connectivity, the uniformity of pore distribution, the twist of connected channels, and the specific surface area of the scaffold. Scaffolds with high porosity and large specific surface area promote seed cell adhesion and growth, extracellular matrix deposition, nutrient and oxygen entry, metabolite discharge, and the ingrowth of blood vessels and nerves [20].

The ideal pore diameter of scaffold materials should be similar to the size of the normal bone unit (the average size of the human bone unit is about 223 μm). Generally, a scaffold pore diameter ranging between 200 and 400 μm is considered adequate [21].

To maintain the desired shape and mechanical strength, the porosity of bone scaffolds should be as high as possible. Ideally, the porosity of the three-dimensional structure should be more than 90%, to successfully promote cells adhesion and growth, bone ingrowth into the material, the transport of nutrients and discharge of metabolites [22,23].

### 2.4. Mechanical Performance

Considering the scaffold as the load bearing implant, the elastic stiffness, strength, and toughness are particularly important [24,25,26]. Too much stiffness in the bone scaffolds results in a high stress shielding phenomenon. The load cannot be transferred from implant to adjacent bone tissue very well, causing filled autogenous bone and the original cancellous bone to not get sufficient mechanical stimulation. After this, bone tissue absorption occurs, leading to the loosening of the implant, ultimately resulting in the failure of bone repair and reconstruction [9,10]. On the contrary, if the stiffness is too low, it reduces the carrying capacity, making the bone more prone to fracture, and thus, the scaffold would not meet the mechanical strength requirements [27].

In addition, the material should be easily machined and retain its shape in the body over time. This implies that the bone biomaterials should have good plasticity [8].

Different materials, designs, and processes have different effects on the mechanical performance of the scaffolds. These effects are discussed further in the following sections.

## 3. Bone Scaffold Materials: Classification and Development Trend

Bone scaffold materials can be divided into two categories: biodegradable and non-biodegradable. The early scaffold materials were non-biodegradable. These materials are characterized by high mechanical strength, good resistance to wear, fatigue and deformation, and biological inertness (resistant to acids and alkalis, anti-aging, no degradation). Nevertheless, non-biodegradable materials cause secondary surgery problems, hence the increased interest in the use of biodegradable and biologically active materials. At present, most of the bone tissue scaffold materials used for research and treatment are biodegradable or a combination of biodegradable and non-biodegradable materials [28].

### 3.1. Metal Materials

Metal materials were one of the earliest biological materials developed for human use. They were widely used in artificial prostheses, artificial joints, and medical instruments, and so on. Metal materials are characterized by high strength, fatigue resistance, and easy processing, and some materials also have significant toughness. Therefore, metal materials still play an important role in the regeneration of bone, teeth, and other parts, which bear high loads [29].

At present, the most commonly used metal materials are stainless steel, cobalt-based alloy, and titanium-based alloy.

The main problem of metal materials is the spread of metal ions to surrounding tissues, caused by the corrosion induced by the physiological environment. This may lead to toxicity and unwanted side effects. In addition, the difference between Young’s modulus of the bone and that of the implant can cause the so-called ‘stress shielding’, which can subsequently inhibit the transfer of physiological stress to bone tissues [30]. To solve these problems, researchers use various surface modification techniques to improve the biocompatibility of the material [31]. This can reduce complications and prolong the service life.

### 3.2. Non-Metallic Inorganic Materials

Natural inorganic materials are mainly coral materials and microwave sintered cuttlefish bone. Calcium carbonate is the main component of coral, which has the advantages of high porosity and good osteoconductivity, as well as being abundant in nature. However, its disadvantages include poor mechanical properties and poor osteoinductivity, limiting its application in bone tissue engineering. Microwave sintered squid bone is a porous, pure bone mineral material, obtained by calcination, which is the heating of the material at high temperatures. After being microwave sintered, the organic matter can be removed from the squid bone, while the antigenicity and bacteria are completely inactivated. At the same time, the high porosity of the bone mineral and the three-dimensional pore structure suitable for osteogenesis, can be preserved [32]. It has good biocompatibility, so MMSCs can adhere to the surface cells and grow well. Moreover, this material promotes stem cell osteogenic differentiation to a certain extent.

The synthetic inorganic materials used in bone tissue engineering are mainly bioceramics, which are nonmetallic materials processed by high temperature treatment. Among them, HA and tricalcium phosphate have been more intensely studied. Inert bioceramics (such as alumina bioceramics) react very little with human tissues in vivo and can be used in artificial joints. Bioactive bioceramics, such as calcium phosphate-based bioceramics, can form chemical bonds with the tissue interface in the physiological environment, resulting in osseointegration. Furthermore, bioactive bioceramics, such as HA, bioactive glass, and bioactive glass ceramics, are used for prosthesis surface coating and as artificial bone grafting materials [33]. Bioabsorbable/biodegradable bioceramics, such as tricalcium phosphate, can gradually degrade in vivo and then be absorbed by the bone tissue. Calcium phosphate cement is another example of biodegradable ceramics, which has multiple applications as a scaffold material.

### 3.3. Organic Polymer Materials

Biomedical organic polymer materials, with relative molecular masses ranging from tens of thousands to several millions, are often used for human organs, tissues, joints, and as drug carriers. Of these, polyglycolic acid, polylactic acid, and the polyglycolide copolymers are the most widely studied.

According to their origin, biomedical organic polymer materials can be divided into two types: natural and synthetic.

Natural organic polymer materials, like collagen, has good biocompatibility, causing no irritation or toxicity after implantation in the human body. It can promote cell proliferation and accelerate wound healing. Furthermore, collagen is biodegradable and can be absorbed by the human body. Its degradation products are non-toxic and cause no unwanted side effects. Fibrin, mainly from plasma proteins, has good blood and tissue compatibility, and causes no toxicity or other adverse effects. It has been clinically used for a long time, typically in filling bone defects after surgery or in chronic osteitis, osteomyelitis, and arthroplasty [34].

Synthesis of polymer biomaterials, like polylactic acid, does not stimulate the tissue, and resulting materials have a bending strength of more than 120 MPa. Moreover, organic polymers can also be used as absorbable internal fixation materials. For example, polylactic acid is eventually metabolized into carbon dioxide, water, etc., which can be completely absorbed by the human body. This means that it can be implanted into the body, without having to be removed later. It is suitable for bone (segment) fixing after a cancellous bone fracture, osteotomy, or arthrodesis/joint fusion [35]. PMMA, with good biocompatibility, aging resistance, and high mechanical strength, can be used as bone cement.

### 3.4. Biological Composite

**Figure 3:**
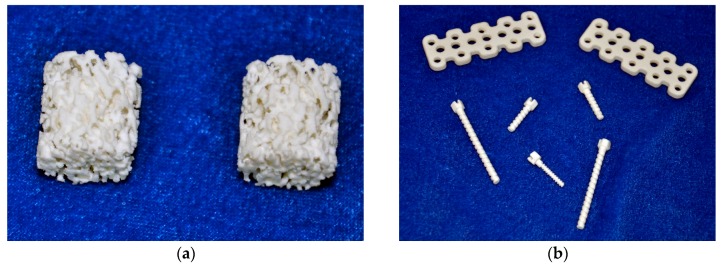
Hydroxyapatite/collagen composite’s application in repairing of bone defects. (**a**) Bone scaffolds of the hydroxyapatite/collagen composite; (**b**) Bone nails and bone plates of the hydroxyapatite/collagen composite.

Due to their good biocompatibility and biodegradability, HA and collagen have become important natural materials for scaffold research. However, certain disadvantages have limited their clinical applications. If special methods are used to combine these two materials, at a certain ratio, into a composite material, it is possible to optimize their biological performance [36,37]. Yanen Wang and Kikuchi [38] etc., have successfully synthesized a hydroxyapatite/collagen composite (Figure 3), which can be successfully involved in bone reconstruction, as it can be replaced by new bone.

Chitosan-acellular dermal material is another material with good biocompatibility. Moreover, it meets the basic requirements of new scaffold materials in tissue engineering, such as good affinity for cells and the abilities to promote cell adhesion, proliferation, and differentiation [39].

### 3.5. Development Trend of Biomaterials

The main trend in the development of biomaterials is to improve the biocompatibility of materials and to develop new materials that are highly biocompatible and more responsive to human physiological needs.

Numerous studies focus on the development of materials by synthesizing extracted natural components into polymers. Also, individual scaffold materials can now be developed into both composite and surface modification materials [40]. Scaffolds generated using additive manufacturing (AM) techniques, typically consist of a biomaterial made of ceramic, metal, self-assembly peptides, synthetic polymers, or natural polymers [41,42,43]. The use of composite materials/composite scaffolds overcomes the specific disadvantages of each material, resulting in a better overall performance. Therefore, composites are becoming increasingly more common [3].

Nanomaterials have unique properties, such as small size effect, interface effect, and interaction between nanostructure units. As nanomaterials are prepared at atomic level, their biggest feature is high specific surface area and porosity, which aid cell inoculation, migration, and proliferation. The strength, toughness, hardness, and superplasticity of nano-ceramic materials have been significantly improved over time. The biomimetic microenvironment of nanofiber materials can affect the interactions between cells, or between cells and scaffold, as well as regulate the biological behavior of the cells. However, the scientific evaluation of the safety performance of nanomaterials is still challenging, limiting their clinical application [33].

To virtually achieve the functions of an artificial extracellular matrix (ECM), the scaffold is often built with interwoven fibrous and porous structures. This can be achieved through the manufacturing processes, which include self-assembly, phase separation, and electrospinning. Among them, electrospinning is the simplest and most versatile. Electrospun nanofibers have many advantages, such as high specific surface area, high porosity, and tunable mechanical properties [44,45]. Most electrospun scaffolds containing nanocarbons, such as carbon nanotubes (CNTs) or graphene-based nanomaterials, are good substrates for the adhesion, growth and differentiation of cells [46]. CNTs, long and slender fullerenes [47], or graphene, have often been used with a wide range of polymers, resulting in a remarkable enhancement of mechanical and electrical properties [46]. In response to the cytotoxicity of CNTs, the introduction of hydrophilic moieties or even bioactive compounds could remarkably reduce the associated risks [48]. Due to its superior biocompatibility, graphene oxide (GO) is the most widely used graphenic compound [46]. Using electrospinning, Wu et al. successfully fabricated nanofibers with incorporated nano graphene oxide (nGO). The resulting material has several advantages, including improved electrospinnability and thermal stability, as well as osteo-bioactivity [49]. Furthermore, Scaffaro et al. proposed important potential applications of biopolymer/nanocarbons scaffolds prepared by electrospinning, as they combine the features of the matrix with those determined by the nanocarbons [46].

Functional materials have special functions under physiological conditions, such as shape memory materials, tissue-guided regeneration materials, surface-modified materials, and so on. Synthetic polymer materials or ceramic materials are modified with bioactive substances, so that the materials, which are inherently unable to induce tissue regeneration, can be changed into regenerative medical materials [50].

The anisotropic feature determines the different mechanical performance of bone tissue, depending on the different portion of stress distribution in vivo [51]. To construct appropriate anisotropic bone tissue microstructures, it is essential to control the morphology and alignment of osteoblast cells [52]. The cells attach and spread along surface grooves and ridges of the scaffolds, which is determined by protein adsorption on the substrate surface [53]. Therefore, the anisotropy and the nano-structure of scaffold materials may influence osteoblast deposition and orientation, subsequently influencing the formation of anisotropic bone tissue [54]. Depth and width are two key factors that determine cell orientation, so that bionic design and optimization of the groove are the most important concerns and hot issues in the study of scaffold surface modification.

Intelligent materials can imitate the life system and have a dual function, of perception and actuation. Perception, feedback, and response are the three key elements of intelligent materials. Traditional materials, combined with high-tech sensors and actuators, are endowed with better performance and more biological properties.

## 4. Bionic Design of Scaffolds for Bone Reconstruction

Bionic design in tissue engineering is mainly used for preoperative diagnosis models and customized bone scaffolds.

Preoperative diagnosis models can help surgeons to define the extent and type of bone defects. As a physical model for preoperative design, recovery simulation, implant choice, and three-dimensional (3D) spatial data measurements, the 3DP preoperative diagnosis model can substantially improve the outcome of surgery, decrease surgery time, and consequently, decrease the possibility of intraoperative and post-operative complications [10].

Customized bone scaffolds provide a living space for the adhesion and proliferation of seed cells. Moreover, these scaffolds support cellular nutrition and metabolism, and provide the necessary mechanical support for the tissue during the bone formation phase [20].

The bionic design of bone scaffolds includes the bionic structure design and bionic performance design. In TE, it is generally accepted that the bionic design determines mechanical properties, and mechanical properties determine cell growth. To a great extent, bionic design determines whether the biological structures prepared in vitro can survive in vivo, as well as whether the surviving bone tissue has proper biological functions. Therefore, bionic design has become a key scientific problem in TE.

### 4.1. Bionic Structure Design

Bionic structure design refers to both shape bionic design and microstructure bionic design.

#### 4.1.1. Shape Bionic Design

For patients with bone defects, the traditional repair methods are bone grafting and general bone implants. However, there are some problems with bone grafting, such as poor plasticity, the difficulty in correcting deformity, and the high incidence of complications, etc. General bone implants should be adjusted during the operation, according to the needs of the repair and the experience of the surgeons, which sometimes results in inaccurate matches with the patient’s own bone. If the adjustment is not properly made, it can affect the appearance, reduce the carrying capacity, as well as cause inaccurate positioning and unstable connections, etc. The basic way to solve these problems is to tailor the bone implants according to the needs of individual patients [27,55].

The bionic design of the bone shape was derived from bionics. Due to the aforementioned disadvantages, surgeons have been working on the creation of prosthesis with similar shapes and structure to the human bone. However, it is still a great challenge to make bone scaffolds with complex shape structures, using traditional technology. Nevertheless, with the development of bionics, the bionic design of bone tissue structure has matured [10].

At present, bone shape bionic design files are mainly obtained through CT image data files, using a special 3D reconstruction software. Although clinicians can digitally reconstruct the model and simulate the surgical procedure, they still need to repair the focus by constant visual inspection and by matching implants in shape. This prolongs the wound exposure time and increases the risk of complications.

In other words, digital orthopedic preoperative planning also depends on the surgeon’s experience. Therefore, preoperative planning using a solid model has great clinical value. To design and create a solid exterior structure, consistent with the shape of the replaced organ, could provide reliable evidence for clinicians to establish a surgical procedure and to plan a fixed route for the implant. Moreover, a solid model can effectively shorten the wound exposure time and duration of surgery, reduce the intraoperative and postoperative complications, and promote patient recovery [10].

#### 4.1.2. Microstructure Bionic Design

At present, the study of bone scaffold materials is quite common. However, in addition to the materials, the porous structure also has a significant impact on scaffold strength, permeability, and cell activities. Therefore, it is important to design suitable micro-bionic structures within the bone scaffolds and to study their effect on performance.

**Figure 4:**
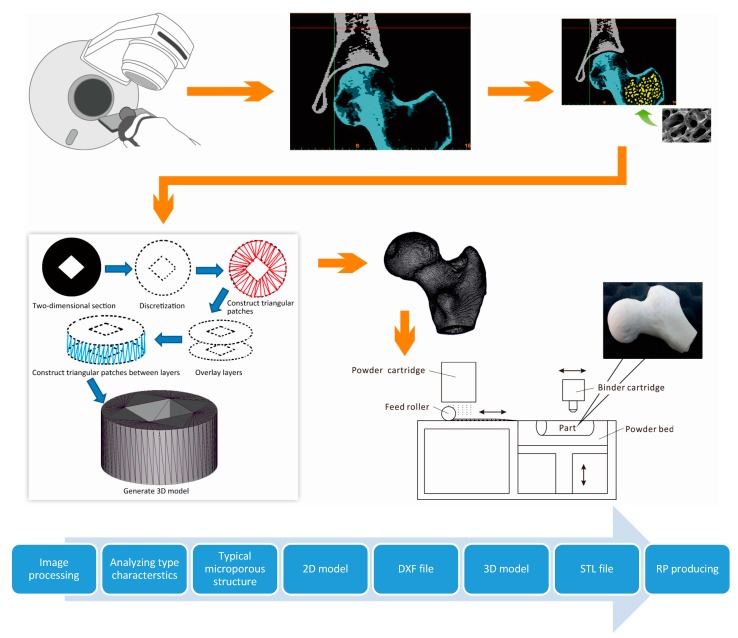
Bionic design and manufacturing process of the inner microstructure of artificial bone scaffold.

With the development of bionics, TE scientists not only imitate the biological shape and function, but also create various microscopic structures within the scaffold. Also, with the bionic design of bone, the internal microstructure of the model is not only similar to the natural cancellous bone, but it also has the function of blood storage and weight reduction. This microstructure also has an important influence on the mechanical properties of the product [56,57]. The honeycomb-like microstructure can serve as a place for bioactive bone cell growth. As the bone grows, the biodegradable material in cancellous bone scaffolds is gradually degraded and replaced by bone macrophages and osteoblasts. To solve the differences in bone structure between individuals, the schematic diagram for bionic design and rapid prototyping of the inner microstructure of artificial bone scaffolds are shown in Figure 4.

In order to fabricate porous scaffolds, facilitating cell incorporation, the AM technique is applied to the biofabrication process, to create living cell/biomaterial/biomolecule constructs [58]. This process is known as Bio-Additive Manufacturing (BAM).

Different from previous construction methods [59], BAM uses computer aided design technology to realize the customized manufacture of the bone defect [60], with an internal 3D-structure that is also similar to the human bone. Therefore, BAM represents an important step forward in bone tissue-engineering [61,62,63].

Over the last two decades, BAM has made significant progress in the fabrication of biomaterials and TE constructs. Until now, the most utilized methods for the fabrication of bone tissue scaffolds continue to be Fused Deposition Modeling (FDM), Stereo Lithography Apparatus (SLA), Selected Laser Sintering (SLS), and 3D Powder Printing (3DP).

### 4.2. Bionic Performance Design

The overall performance of bionic bone scaffolds, after being implanted into the human body, can be assessed based on both mechanical performance and biological performance. The mechanical performance refers to the structural support of bionic bones and matching the stress caused in the host. The biological performance is mainly determined by osteoinductivity, osteoconductivity, and the consequent osteogenesis.

#### 4.2.1. Bionic Design of Mechanical Performance

Multiple studies have shown that the response of the host to the implant is largely determined by the mechanical properties of the scaffold. Biomechanical requirements include adequate static strength (e.g., bending, compression, tensile, shear, etc.), appropriate elastic modulus and hardness, resistance to fatigue, friction, wear, and so on [64].

For the bone model used in preoperative diagnosis, the approximation of bionic design in mechanical properties directly influences the preoperative preparation and the planning of the implants. The bionic preoperative diagnosis model is closer to the real bone in both structure and texture. Also, the mechanical properties have the mechanical gradient effect according to the characteristics of cortical bone and cancellous bone [65].

For artificial bone scaffolds, it is necessary to match the mechanical strength between the artificial substitute and the tissue surrounding the implant site, to ensure the stability and integrity of the scaffold in the in vivo mechanical environment [10], as well as to improve the mechanical function of the patient’s own bone [30].

To better match the mechanical and biological performance with the original bone, the importance of functionally graded scaffold (FGS), made of porous biomaterials, has been increasingly realized in recent years [66]. It possesses the characteristics of complex gradient porosity and function and could mimic the shape, morphology, and overall physiology fully [67]. In the architecture of FGS, porosity, pore size, and pore interconnectivity is of critical importance. Simoneau et al. have studied the mechanical performances of scaffolds with various porosities [68]. Harrysson et al. and Arabnejad et al. have studied the mechanical performances of some functionally graded implants [66,69].

Bone tissue has a highly characteristic anisotropic structure, from nano- to macroscale, which governs its mechanical properties [51,52]. In many cases, the anisotropy is determined by the orientation of the crystallographic texture in the stress direction. To determine the anisotropy and the degree of bone regeneration, special methods have been designed to analyze crystallographic texture and related mechanical performance of the scaffold. Nakano et al. believe that analyzing the orientation distribution of biological apatite (BAp) crystallites by microbeam X-ray diffractometer is “an effective means of evaluating the degree of microstructural regeneration, and also the related mechanical function” [70].

Apart from the material required, the bionic bone fabrication process and surface modification are important factors that restrict the use of artificial scaffolds consistent with the real bones. The maturing of the AM technology will assist in optimizing the design of FGS, and the geometrical structure can be precisely controlled. Therefore, the mechanical and biological performances will match with the original bone better through FGS [67] Furthermore, Noyama et al. optimized the implant design for long-term fixation, by creating the ‘oriented groove’ as a potent surface modification [30].

#### 4.2.2. Bionic Design of Biological Performance

To achieve good biological performance, bionic bone scaffolds in vivo should produce no harmful degradation products, be resistant to body fluid erosion, and experience no water absorption, swelling, softening, or deterioration.

Furthermore, the ideal bionic bone material should have good bioactivity, biodegradability, and osteogenesis. As previously mentioned, bioactive materials can exchange substances with human bones, biodegradable materials can be gradually replaced by human bones, and osteogenic materials can transfer the force of bones and act as a mechanical support to avoid stress concentration, caused by the high mechanical strength of the material. Simultaneously satisfying these requirements would lead to proper bone absorption.

Due to intensive research, many biologically porous materials were developed as scaffold carriers, to construct TE bone. Different growth factors were loaded onto these scaffolds, to help repair and reconstruct bone defects. This research field has become a hot topic in recent years [71].

## 5. Summary

At present, multiple experimental validations of scaffold materials and processing methods have proved to greatly promote the development of bone tissue engineering. However, the ideal bone tissue engineering requirements are yet to be met, as there are still some problems to be solved.

### 5.1. Combining Metal Materials and Other Materials to Form New Composite Materials Is a Growing Trend

Different materials have different mechanical and biological performances, and each have their own advantages and disadvantages. The poor biocompatibility and degradation are urgent problems to be solved for metal materials. Meanwhile, polymer materials have poor mechanical strength. Some polymers may have incomplete antigenic elimination, or cause cell poisoning due to organic solvent residue, as well as many other issues. For inorganic materials, the main problem that needs to be solved is the inability to control their mechanical properties and degradation rate.

Comprehensive consideration should be given to the advantages and disadvantages of inorganic materials and organic polymer materials, especially when combining metal materials with non-metallic inorganic materials. Various materials complement each other to form new composite materials or new composite scaffolds. This is done in an attempt to meet the requirements of the ideal scaffold for bone tissue engineering.

### 5.2. Good Mechanical and Biological Performances Must Be Simultaneously Achieved

There is currently a contradiction between the strength of the bone scaffold and its porosity. An increase in porosity can improve the adhesion of osteoblasts and the biological properties of the implants, but at the expense of reducing the mechanical properties of the scaffolds.

The biological properties of the metal scaffolds can be improved via surface modification, which can enhance the adhesion of osteoblasts to the scaffold material and ensure good mechanical properties. The most commonly used coating material for surface modification is HA. Hydroxyapatite and titanium alloy bonds are powerful, being able to not only form a mechanical combination, but also a chemical combination [31]. Theoretically, an HA/titanium composite is an ideal solution.

### 5.3. Bionic Design Is an Effective Way to Find a Balance Between Mechanical Performance and Biological Performance

Bionic design and scaffold preparation techniques play key roles in the modification of materials. To improve the characteristics of scaffold materials, in hopes of satisfying the mechanical and biological performance requirements of bone scaffolds, bionic design, BAM, nano-technology, controlling material porosity, and surface modification of scaffold materials can be used.

The main research objective for the future is to develop scaffold materials with stable performance, easy access, easy processing, and with similar structure and properties to those of the human bone. Another objective is to use bionic design, in trying different combinations of materials, to develop the ideal composite scaffold for bone TE. This should have good biocompatibility, controllable degradation, proper mechanical strength, and support surface adhesion and induction of seed cells.
